# Two-Year Experiences of 500 Appendectomies in Lahore General Hospital, Lahore

**DOI:** 10.7759/cureus.21303

**Published:** 2022-01-16

**Authors:** Muhammad H Shahid, Faisal I Khan, Zain A Askri, Arslan Asad, Rabia Saeed, Talha B Talib, Anwar Z Khan, Tausief Fatima, Muhammad F Afzal

**Affiliations:** 1 General Surgery, Lahore General Hospital, Lahore, PAK; 2 General Surgery, Fatima Memorial Hospital, Lahore, PAK

**Keywords:** perforated appendix, laparotomy, laparoscopic appendectomy, open appendectomy, appendicitis

## Abstract

Introduction

Acute appendicitis is a leading cause of abdominal conditions in emergency departments. Evidence from research studies has indicated the efficacies of surgical procedures involving appendectomies. However, in Pakistan, there is a paucity of information regarding the epidemiology, clinical presentations, and surgical management of acute appendicitis.

Objective

This paper aims to report the epidemiologic data and findings of surgical management of acute appendicitis in Lahore General Hospital (LGH). The data was based on our two-year experiences of appendectomies in the hospital.

Materials and methodology

Data were collected retrospectively. The patients underwent appendectomies performed by the team of surgeons of Surgical Unit 1 of LGH in the Accident and Emergency (A&E) Department from July 2019 to October 2021.

Results

The total number of patients was 506, and the mean age was 26.8. Males (67.29%) and young adults aged 18-34 years were at higher risk of appendicitis. Compared to other surgical procedures performed, open appendectomy and laparoscopic appendectomy operative times were significantly shorter. Histopathology of all the cases showed acute inflammation of the appendix.

Discussion

Similar to findings from other research studies, the operative time of open appendectomies was shorter (70.6 minutes) in the hospital than the operative time of laparoscopic appendectomies (77 minutes). However, the overall operative times were longer than the operative times reported in some other research studies. Also, contrary to other research findings, open appendectomy (1.22 days) was associated with a longer length of hospital stay than laparoscopic appendectomy (≈1 day). Simple acute appendicitis was the most predominant operation findings (289, 57.1%).

Conclusion

Compared to other hospitals, the shorter hospital stays/recovery time indicated the high surgical skill of performing open and laparoscopic appendectomies in Lahore General Hospital, Lahore, Pakistan.

## Introduction

Appendicitis is one of the most predominant causes of acute abdominal cases, and it is responsible for 7%-10% of the total emergency clinical conditions [1]. Acute appendicitis is one of the leading causes of lower abdominal pains that cause patient emergency department visits. Furthermore, it is the most diagnosed abdominal condition in hospitalized patients with acute abdominal cases [2]. Evidence from research studies has indicated geographical differences in the incidence rates of acute appendicitis. In the United States, the reported incidence rate was 9%; in Europe, it was 8%; in Africa, it was 2% [3]. Notable differences can be observed in the presentation of acute appendicitis, its severity, and surgical management according to countries and their economic condition [4]. Perforation rates, for example, range from 16% to 40%, with younger patients (40%-57%) and older patients (55%-70%) being more affected [5]. Also, the incidence rates of acute abdominal pain vary between males and females [6].

Morbidity and mortality are significantly increased with perforated acute appendicitis compared with nonperforated appendicitis. Furthermore, evidence from research studies indicated higher risks of mortality with gangrenous acute appendicitis [7]. However, reports from research studies have indicated the efficacy of the surgical intervention, via appendectomies, in mitigating the high morbidity and mortality rates associated with perforated appendicitis [8]. Addiss et al. (1990) reported that over 300,000 appendectomies are performed yearly in the United States [9]. However, in Pakistan, there is a paucity of published information regarding the epidemiology, clinical presentations, and surgical management of acute appendicitis. Hence, this paper aims to report our two-year experiences in performing appendectomies as surgeons in Lahore General Hospital (LGH), Lahore, Pakistan.

## Materials and methods

This retrospective cross-sectional study was carried out over a 27-month period from July 2019 to October 2021, and patients were surgically managed in the Accident and Emergency (A&E) Department of Lahore General Hospital. Patients with the clinical diagnosis of acute appendicitis were included, and patients whose appendectomy was conducted by other surgical departments of LGH were excluded. Patient records and surgery notes were used to compile the data. The patients in the study ranged in age from 15 to 68 years old.

A detailed history of the start of discomfort, radiation, anorexia, vomiting, and fever was taken. A complete description of menstrual history was noted in females of childbearing age (14-44 years) to rule out pelvic inflammatory illness. To rule out ureteric colic, all male patients with right iliac fossa discomfort and a history of burning sensation during micturation and/or hematuria were examined. A general survey was conducted, with a focus on measuring pulse, temperature, and blood pressure. McBurney’s point, psoas test, obturator test, cough sign, pain on straight leg raise, localized stiffness of the right iliac fossa, and rebound tenderness were all part of the abdominal examination. Every patient has to undergo a rectal examination. To look for symptoms of sepsis, other systems were checked. After provisionally diagnosing the patient with appendicitis, additional tests to confirm the diagnosis included a total count to check for leucocytosis; a biochemical examination to check for blood sugar, urea, and creatinine; an upright X-ray abdomen; and ultrasound. For all patients with appendicitis, a final decision on operational intervention was taken.

Open explorations and laparoscopic appendectomies were used to perform appendectomies. Due to advanced illness levels, several patients from the laparoscopic method were switched to open exploration when needed.

Open appendectomy was performed through a Lanz or gridiron incision. Some patients needed lower midline laparotomy as per requirement. For laparoscopic appendectomy, three ports (umbilical (10 mm), suprapubic (5 mm), and left iliac fossa (10 mm)) were performed. The appendicular artery was dissected and divided between hemostatic energy devices. The appendix was secured at the base with three loop ligatures, divided between the two distal ligatures, and removed through the 10-mm umbilical port.

If there were technical difficulties or an advanced stage of infection, such as four quadrant pus, the laparoscopy was modified to an open appendicectomy or lower midline laparotomy. Histopathological evaluation of the resected appendix was performed.

In patients with uncomplicated appendicitis, intravenous fluids (IVFs) were maintained for six hours following surgery, and a regular diet was resumed after that. IVF was maintained in severe instances (patients with perforation and peritonitis) until the normal intestinal function was restored (return of bowel sounds and passage of flatus). For simple instances, antibiotic prophylaxis consisted of a single dose of a third-generation cephalosporin. Meronem was administered preoperatively with metronidazole at induction and again after 12 hours in difficult patients.

Analgesics were administered in the form of ketorolac injections for a period of 24 hours. Additional analgesics were prescribed depending on the patients’ pain perception.

The operating time and the length of hospital stay were recorded, as in comparable series. The patients were encouraged to return to their regular activities and work as soon as they felt ready. Normal activity was defined as the patient’s return to normal household and social activities of his or her choosing.

For one month, the patients were followed up on weekly basis, but none of the patients required readmission. The Statistical Package for the Social Sciences (SPSS) version 24 for Windows was used to statistically evaluate the data obtained.

## Results

Demographics

The total number of patients diagnosed with appendicitis and who underwent appendectomies in the Surgical Unit of Lahore General Hospital was 506. The mean age was 26.58 ± 7.24 years. Seventy-three (14.43%) of the patients were 17 years and younger, 341 (67.29%) were 18-34 years, 60 (11.86%) were 35-44 years, 25 (4.94%) were 45-65 years, and seven (1.38%) were 65 years and older. There were 310 (61.3%) males and 196 (38.7%) females.

Procedures performed

Five surgical approaches were opted for managing acute appendicitis, depending upon the operative findings, during the period. The surgical procedures performed are simple open appendectomy, laparoscopic appendectomy, laparoscopy converted to midline laparotomy, open appendectomy converted to midline laparotomy, and lower midline laparotomy. Two hundred twenty-seven (54.7%) patients underwent open appendectomy, 164 (32.4%) underwent laparoscopic appendectomy, 34 (6.7%) underwent laparoscopy converted to midline laparotomy and appendectomy, 20 (4%) underwent open appendectomy converted to midline laparotomy, and 11 (2.2%) underwent lower midline laparotomy (Table 1).

**Table 1:** Procedures performed with the number of patients

Procedure	Frequency (N)	Percentage (%)	
Open appendectomy	227		54.7
Laparoscopic appendectomy	164		32.4
Laparoscopy converted to midline laparotomy and appendectomy	34		6.7
Open appendectomy converted to midline laparotomy	20		4
Lower midline laparotomy	11		2.2
Total	506		100

Procedures Performed Versus Duration of Operation

The mean duration of the procedures performed was as follows: open appendectomy, 70.6 minutes; laparoscopic appendectomy, 77 minutes; laparoscopy converted to midline laparotomy and appendectomy, 139.4 minutes; open appendectomy converted to midline laparotomy, 146.75 minutes; and lower midline laparotomy, 139 minutes (Table 2). The correlation between the surgical procedure performed and operative time was positive (0.63). Statistical analysis of the effect of surgical operation on the operative time indicated that the operative time was significantly dependent on the type of surgical procedures performed (p ≤ 0.001).

**Table 2:** Mean duration of the procedures performed

Procedures performed	Mean duration of operation (minutes)
Open appendectomy	70.60
Laparoscopic appendectomy	77.04
Laparoscopy converted to midline laparotomy and appendectomy	139.41
Open appendectomy converted to midline laparotomy	146.75
Lower midline laparotomy	139.09

Procedures Performed Versus Duration of Hospital Stay

The median duration of patient hospital stay after performing open appendectomy was 1.22 days, laparoscopic appendectomy was 1 day, laparoscopy converted to midline laparotomy and appendectomy was approximately 4.7 days, open appendectomy converted to midline laparotomy was 4.6 days, and lower midline laparotomy was 5.45 days (Table 3). The correlation between the surgical procedure performed and the duration of hospital stay after the surgical procedure was positively strong (0.75) and statistically significant (p ≤ 0.001) (Table 3). Statistical analysis of the effect of surgical operation on hospital stay indicated that the duration of hospital stays was significantly dependent on the type of surgical procedures performed (p ≤ 0.001).

**Table 3:** Mean duration of hospital stay for each surgical procedure performed

Procedures performed	Mean duration of hospital stay (days)
Open appendectomy	1.22
Laparoscopic appendectomy	1.09
Laparoscopy converted to midline laparotomy and appendectomy	4.65
Open appendectomy converted to midline laparotomy	4.60
Lower midline laparotomy	5.45

Surgical operation findings

The findings from the surgical operation included acute appendicitis, acute gangrenous appendicitis, appendicular mass, appendicitis with minimal pus formation, appendicitis with four quadrant pus, perforated appendix with localized pus formation, and a perforated appendix with four quadrant pus formation. Simple acute appendicitis was found in 289 (57.1%) patients, acute gangrenous appendicitis in 32 (6.3%) patients, appendicular mass in 38 (7.5%) patients, appendicitis with minimal pus formation in 55 (10.9%) patients, appendicitis with four quadrant pus in 24 (4.7%) patients, perforated appendix with localized pus formation in 27 (5.3%) patients, and perforated appendix with four quadrant pus formation in 41 (8.1%) patients (Table 4). Ten (2%) patients had ovarian cysts, while two patients (0.4%) had pelvic inflammatory disease. No mortality was recorded.

**Table 4:** Surgical operation findings with the number of patients

Findings	Frequency (N)	Percentage (%)
Acute appendicitis	289	57.1
Acute gangrenous appendicitis	32	6.3
Appendicular mass	38	7.5
Appendicitis with minimal pus formation	55	10.9
Appendicitis with four quadrant pus	24	4.7
Perforated appendix with localized pus formation	27	5.3
Perforated appendix with four quadrant pus formation	41	8.1
Total	506	100

Operation Findings by Age Group

The young adults, aged 18-34 years, showed the highest number of all the operation findings, while the operation findings were most minimal in the older adults, 65 years and older. In all age groups, except the older adults (65 years and older), acute appendicitis is the most frequently observed surgical finding (Table 5). The t-test indicated that there were statistically significant differences in the surgical operation findings between the age groups (p ≤ 0.001). The correlation between the age and operation findings was positive, but it was weak (0.084).

**Table 5:** Surgical operation findings by age groups

Age (years)	≤17	18–34	35–44	45–64	≥65
Acute appendicitis	53	197	25	11	3
Acute gangrenous appendicitis	2	22	3	5	0
Appendicular mass	3	25	4	2	4
Appendicitis with minimal pus formation	12	30	10	3	0
Appendicitis with four quadrant pus	0	18	6	0	0
Perforated appendix with localized pus formation	0	20	7	0	0
Perforated appendix with four quadrant pus formation	03	29	5	4	0

Operation Findings Versus Duration of Operation

The duration of performing appendectomies with acute appendicitis was 69.33 minutes, with acute gangrenous appendicitis was 78.59 minutes, with appendicular mass was 67.24 minutes, with appendicitis with minimal pus formation was 79.18 minutes, with appendicitis with four quadrant pus was 149.38 minutes, with perforated appendix with localized pus formation was 101.11 minutes, and with perforated appendix with four quadrant pus formation was 137.07 minutes (Table 6). Statistical analysis indicated that the operative time was significantly dependent on the type of operation findings (p ≤ 0.001).

**Table 6:** Surgical operation findings and duration of operation

Operation findings	Mean duration of operation (minutes)
Acute appendicitis	69.33
Acute gangrenous appendicitis	78.59
Appendicular mass	67.24
Appendicitis with minimal pus formation	79.18
Appendicitis with four quadrant pus	149.38
Perforated appendix with localized pus formation	101.11
Perforated appendix with four quadrant pus formation	137.07

Operation Findings Versus Hospital Stay

The duration of hospital stay for patients with acute appendicitis was 1.13 days, with acute gangrenous appendicitis was 1.28 days, with appendicular mass was 1.11 days, with appendicitis with minimal pus formation was 1.15 days, with appendicitis with four quadrant pus was 4.71 days, with perforated appendix with localized pus formation was 1.52 days, and perforated appendix with four quadrant pus formation was 4.80 days (Table 7). Statistical analysis indicated that the duration of hospital stays was significantly dependent on the type of operation findings (p ≤ 0.001). Histopathology of all the specimens turned out to be acute inflammation of the appendix.

**Table 7:** Surgical operation findings and duration of hospital stay

Operation findings	Mean duration of hospital stay (days)
Acute appendicitis	1.13
Acute gangrenous appendicitis	1.28
Appendicular mass	1.11
Appendicitis with minimal pus formation	1.15
Appendicitis with four quadrant pus	4.71
Perforated appendix with localized pus formation	1.52
Perforated appendix with four quadrant pus formation	4.80

Appendix position

Six appendix positions were identified in the patients: retrocecal, subhepatic, sub-cecal, post-ileal, pre-ileal, and pelvic. The appendix position was retrocecal in 58 (11.5%) patients, subhepatic in 10 (2%) patients, sub-cecal in 56 (11.1%) patients, post-ileal in 73 (14.4%) patients, pre-ileal in 66 (13%) patients, and pelvic in 243 (48%) patients (Table 8).

**Table 8:** Appendix position with the number of patients

Positions	Frequency (N)	Percentage (%)
Retrocecal	58	11.5
Subhepatic	10	2
Sub-cecal	56	11.1
Post-ileal	73	14.4
Pre-ileal	66	13
Pelvic	243	48
Total	506	100

## Discussion

The most affected patient age group includes the young adults, aged between 18 and 34 years (67.29). A higher percentage of the patients were males (67.29%), indicating that males are at higher risks of acute appendicitis than females. Other research studies have reported similar findings. AlHarmi et al. (2021) found that among 646 patients diagnosed with appendicitis, 500 (77.4%) were males [10]. Research studies that investigated the risk of appendicitis between males and females indicated that males are at a higher risk of developing acute appendicitis than females [11,12]. From 506 appendectomies in the hospital within the period, more than half (227, 54.7%) were open appendectomies, while one-third (164, 23.4%) were laparoscopic appendectomies (Table 1).

Regarding the duration of performing the surgical procedures (operative time), open appendectomy and laparoscopic appendectomy were significantly faster to perform (p ≤ 0.001) than other surgical procedures. Statistical analyses showed that the operative time and hospital stay were significantly dependent on the type of surgical procedures (p ≤ 0.001). The mean duration of performing open appendectomies was shorter (70.6 minutes) than the mean duration for performing laparoscopic appendectomies (77 minutes) (Table 2). Research studies that compared the operative time between the surgical procedures reported the same findings that the duration of open appendectomies was shorter than that of laparoscopic appendectomy [13-15]. Shimoda et al. (2018) noted that the longer operative time in laparoscopic appendectomy was due to the associated complications and largely dependent on the surgeon’s skill and experience [16]. Compared to findings from other research studies, the operative time for the two procedures was longer.

The results of this research study showed that open appendectomy resulted in longer hospital stays (1.22 days) than laparoscopic appendectomy (approximately 1 day) (Table 3). The results contradict the findings from other research studies. The findings from the other research studies indicated that open appendectomy is associated with a shorter hospital stay than laparoscopic appendectomy [13-15]. Furthermore, the operative time for the two surgical procedures was considerably shorter than the operative time reported by other research studies. Biondi et al. (2016) reported 2.7 days for laparoscopic appendectomy and 1.4 days for open appendectomy [13]. Nazir et al. (2019) reported 4.38 days for laparoscopic appendectomy and 4.18 days for open appendectomy [14]. Takami et al. (2020) reported 12.19 days for laparoscopic appendectomy and 9.61 days for open appendectomy [15]. The shorter hospital stay compared to other research findings might be attributed to the better surgeons’ skills and experiences in performing an open appendectomy and laparoscopic appendectomy in Lahore General Hospital, Lahore.

Acute appendicitis was the most predominant operation findings, which is more than half (289, 57.1%) of the findings (Table 4). Many research studies have similarly reported the high incidence and prevalence rates of acute appendicitis in patients with acute abdominal conditions [1,2]. The operation findings vary by age group. The numbers of all the operation findings were significant in young adults aged between 18 and 34 years than other age groups (p ≤ 0.001) (Table 5). This can be attributed to dietary habits. Evidence from research studies has indicated the relationship between the risk of acute appendicitis and dietary habits [17-19]. However, there was no correlation between the operation findings and the age groups, indicating that the operation findings could not be attributed to aging but could be attributed to the age group factor. Appendicitis with four quadrant pus, perforated appendix with localized pus formation, and a perforated appendix with four quadrant pus formation was associated with significantly longer operative time than other operation findings (Table 6). Appendicitis with four quadrant pus and a perforated appendix with four quadrant pus formation resulted in significantly longer hospital stays (Table 7). The majority of the patients (243, 48%) had their appendix located in the pelvic region (Table 8).

Limitations

This is a single-center study and personal experience of appendectomy performed by surgeons of Surgical Unit 1 of Lahore General Hospital.

## Conclusions

Based on the findings of this research study, young adults (18-34 years) and males are at the highest risk of developing acute appendicitis. Surgeons in Lahore General Hospital, Lahore, are highly skilled in appendectomies, performing the surgical operations in considerably shorter operative time than reported in other hospitals. Contrary to findings from other research studies, the surgeons in the hospital could perform laparoscopic appendectomy in a shorter operative time than open appendectomy. Furthermore, the surgeries were associated with a shorter length of hospital stay than reported in other hospitals. It implies that the surgeons in Lahore General Hospital are highly skilled and knowledgeable in performing laparoscopic appendectomy with considerably shorter lengths of hospital stay.
